# Detection of diabetic retinopathy using a fusion of textural and ridgelet features of retinal images and sequential minimal optimization classifier

**DOI:** 10.7717/peerj-cs.456

**Published:** 2021-05-07

**Authors:** Lakshmana Kumar Ramasamy, Shynu Gopalan Padinjappurathu, Seifedine Kadry, Robertas Damaševičius

**Affiliations:** 1 Hindusthan College of Engineering and Technology, Coimbatore, India; 2 Vellore Institute of Technology University, Vellore, India; 3 Noroff University College, Kristiansand, Norway; 4 Department of Applied Informatics, Vytautas Magnus University, Kaunas, Lithuania

**Keywords:** Diabetic Retinopathy, Fundus image, Textural features, Image processing, Continous Ridgelet transform

## Abstract

Diabetes is one of the most prevalent diseases in the world, which is a metabolic disorder characterized by high blood sugar. Diabetes complications are leading to Diabetic Retinopathy (DR). The early stages of DR may have either no sign or cause minor vision problems, but later stages of the disease can lead to blindness. DR diagnosis is an exceedingly difficult task because of changes in the retina during the disease stages. An automatic DR early detection method can save a patient's vision and can also support the ophthalmologists in DR screening. This paper develops a model for the diagnostics of DR. Initially, we extract and fuse the ophthalmoscopic features from the retina images based on textural gray-level features like co-occurrence, run-length matrix, as well as the coefficients of the Ridgelet Transform. Based on the retina features, the Sequential Minimal Optimization (SMO) classification is used to classify diabetic retinopathy. For performance analysis, the openly accessible retinal image datasets are used, and the findings of the experiments demonstrate the quality and efficacy of the proposed method (we achieved 98.87% sensitivity, 95.24% specificity, 97.05% accuracy on DIARETDB1 dataset, and 90.9% sensitivity, 91.0% specificity, 91.0% accuracy on KAGGLE dataset).

## Introduction

**Figure 1:**
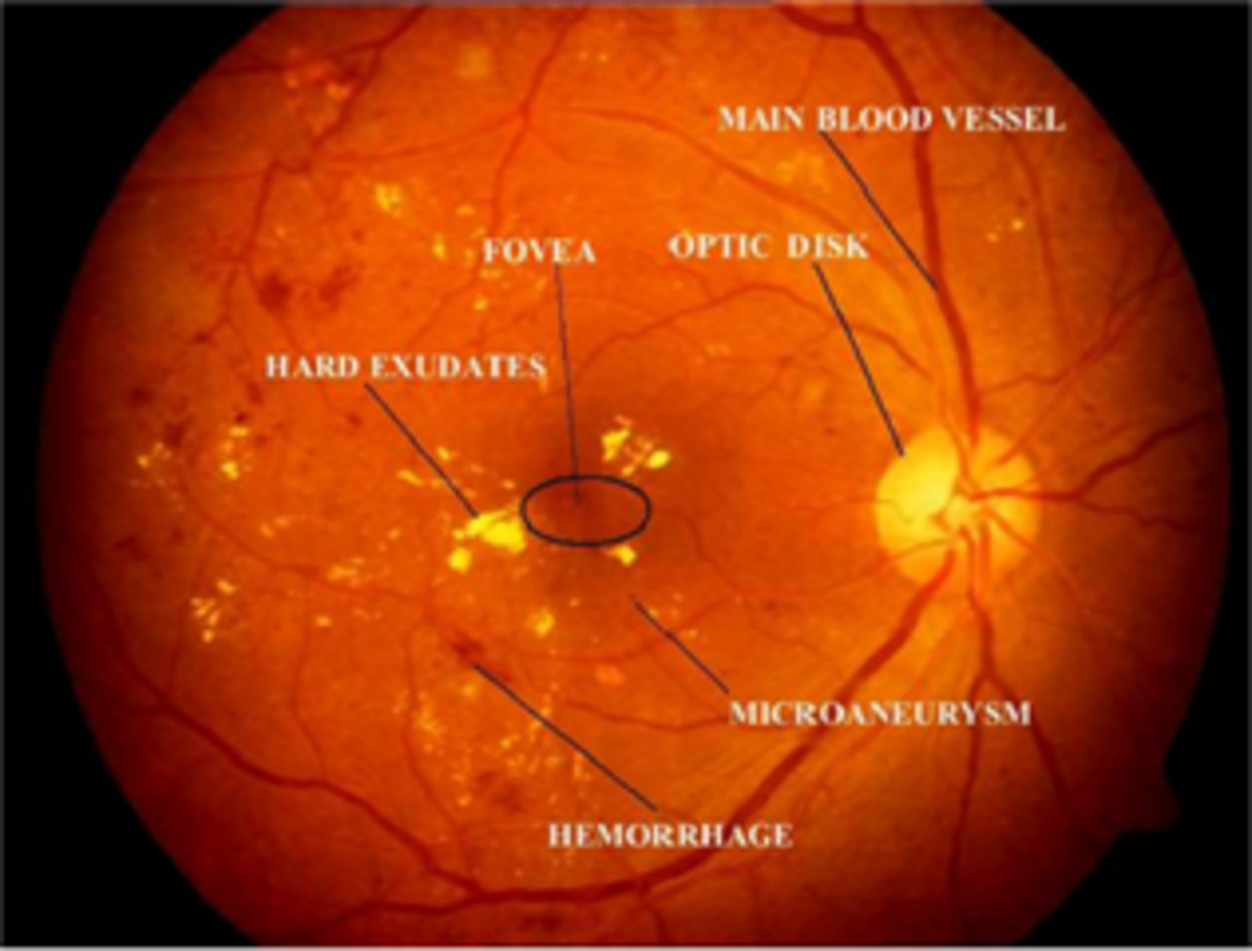
Illustration of retinal image features. Image credit: DIARETDB1. © Tomi Kauppi, Valentina Kalesnykiene, Joni-Kristian Kamarainen, Lasse Lensu, Iiris Sorri, Asta Raninen, Raija Voutilainen, Juhani Pietilä, Heikki Kälviäinen, and Hannu Uusitalo.

The World Health Organization (WHO) assesses that 347 million people currently suffer from diabetes and that in 2030 this disease will be the seventh leading reason for death in the world (Murugan, 2019). Over the years, diabetes patients will usually exhibit deviations from the retina norm, causing an issue called diabetic retinopathy (DR). It is a serious cause of visual loss, including blindness. It involves type 1 and type 2 diabetes complications. DR is caused by impaired blood vessels in the retina. Ophthalmologists diagnosis DR by studying exacting and time-intensive images of the retinal fundus. Automating DR diagnosis will reduce the pressure on ophthalmologists to concentrate on vulnerable patients and allow further medical screening (Umapathy et al., 2019). Retinal lesions such as hemorrhages (HRs), micro-aneurysms (MAs), and hard exudates (HE) can be used to identify DR affected retinal images. Figure 1 shows the retinal features.

**Figure 2:**
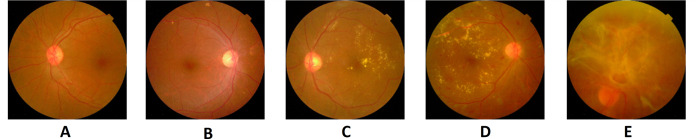
Five stages of diabetic retinopathy in fundus images: (A) without DR, (B) mild, (C) moderate, (D) severe, (E) PDR. Image credit: DIARETDB1. © Tomi Kauppi, Valentina Kalesnykiene, Joni-Kristian Kamarainen, Lasse Lensu, Iiris Sorri, Asta Raninen, Raija Voutilainen, Juhani Pietilä, Heikki Kälviäinen, and Hannu Uusitalo.

There are usually two types of DR, such as Proliferative DR (PDR) and Non-Proliferative DR (NPDR). As new blood vessels develop on the surface of the retina, PDR has the elements of neovascularization and vitreous fluid hemorrhage and can bleed. But there are no signs in NPDR and it can only are identified by the retinal image (Gharaibeh, 2016). The fundus images are graded by the clinical specialist as the normal retina, mildly affected, moderately affected, and severely affected NPDR retina and PDR retina (shown in Fig. 2).

The typical retina image does not display any signs of DR characteristics, including retinal lesions, while blood vessels are clear without any leakage. In the case of mild NPDR, the micro-aneurysms are observed. During the moderate NPDR, there are microaneurysms, hemorrhages, damaged exudates, and blood vessels that can expand and distort. One of the signs will recognize severe NPDR: blockage of certain blood vessels in retinas, development of new blood vessels. The PDR damaged retina has the consequences of forming new blood vessels, which are neovascularization of an abnormal nature. It is developed at the back of the eye; as a result, the vision is blurry; it can burst or bleed (Li & Li, 2013).

Many automatic DR detection techniques (Argade et al., 2015; Chetoui, Akhloufi & Kardouchi, 2018; Lam et al., 2018) were proposed. One of the main challenges with DR diagnosis is that at its early level, it is impossible to recognize the signs. When it goes beyond the advanced level, it can lead to vision failure entirely. There are other approaches developed to diagnose DR and its intensity rating as a problematic activity for its earlier identification. Several strategies were implemented to diagnose the difficulty in its early stages to address this challenge. Having the methods reliable, precise, and cost-effective is incredibly critical.

The principal cause of mortality for people living with diabetes is cardiovascular disease (CVD). While diabetes was previously considered a risk similar to CVD, the variability of this risk is gradually being recognized, with recent guidance available explicitly targeting diabetic patients providing risk evaluation (Ho et al., 2017). The inclusion of retinopathy can mean a more negative cardiovascular risk profile for people with type 2 diabetes. It is understood that diabetics with retinopathy are at risk to have associated cardiovascular disease factors, such as obesity and dyslipidemia, relative to those without retinopathy, which can all increase their risk of cardiovascular disease (Klein et al., 2002). Retinopathy is related to an increased risk of CVD in people with diabetes (Cheung et al., 2013). In addition to these qualitative measures of microvascular pathology, novel indices of microvascular injury such as improvements in the retinal vascular caliber can currently be assessed using computer-assisted systems from the same retinal photographs (Ikram et al., 2013). Research also demonstrates that certain tests of microvascular disruption are consistent with CVD not just in the common people (McGeechan et al., 2008), and in cronies with diabetes. It indicates the retinal tests taken from retinal images, representing systemic properties of microvascular disease.

Retinal fundus images were often used to detect retinal diseases. Diabetic retinopathy disorders are observed by image processing algorithms. For the identification of hard exudates, cotton dots, hemorrhage, and microaneurysm lesions that arise in the initial stages of the disease, an algorithm focused on retinal image processing techniques and a decision support system was built in Akyol, Bayir & Sen (2017). Park & Summons (2018) implemented an effective method for automatically detecting MAs in a retinal photograph. The approach is based on an automated wavelet transformation and the decision tree (C4.5) algorithm, which distinguishes cases of DR and non-DR. In RGB retinal images, this employs both red and green channel data to detect tiny MAs and obtains the image parameters. Random Forest (RF) was used in Roychowdhury & Banerjee (2018) to identify retina anomalies caused by Diabetic Retinopathy. A collection of mathematical and geometric features has been derived from photographs in the archive that represent the various physical manifestations of the disorder. Machine learning classification can aid a physician by providing an idea of the severity of the disease. The work in Bhaskaranand et al. (2016) addressed an automatic DR screening method and extended it to automatic microaneurysm (MA) turnover prediction, a possible DR risk biomarker. The DR testing method systematically investigates color retinal fundus photographs from a patient experience for the specific DR pathologies and gathers the details from all the pictures corresponding to patient experience to produce a suggestion for patient monitoring. The MA estimating method aligns retinal images from a patient’s various experiences, locates MAs, and conducts MA dynamics examination to determine recent, recurrent, and incomplete maps of lesions and predict MA turnover.

A method of identification for the five intensity stages of diabetic retinopathy has been developed in Abbas et al. (2017) without taking preprocessing using features derived from a semi-supervised deep-learning algorithm. The work in Gharaibeh et al. (2018) suggested an efficient form of image analysis to diagnose diabetic retinopathy diseases from photographs of the retinal fundus. The following procedures are used for diabetic retinopathy diagnosis: pre-processing, optical disk identification and elimination, segmentation, and elimination of the blood vessels, removal of the fovea, and isolation of features, feature selection, and classification. In Gayathri et al. (2020), the author focused on extracting Haralick and Anisotropic Dual-Tree Complex Wavelet Transform features from retinal fundus images that can conduct accurate DR classification. This characteristic is based on second-order data, and the positional characteristics in photographs are accurately identified by wavelet transform features.

The work in Wang & Yang (2017) suggested a method of deep learning for the identification of interpretable diabetic retinopathy. The interpretable visual function of the proposed approach is accomplished by introducing the activation of the regression map after the convolutional networks’ global averaging pooling layer. This model will find a retina image's discriminative regions to display a particular area of concern in terms of its intensity level. Gargeya & Leng (2017) built and tested a data-driven, deep learning method as a novel diagnostic tool for DR detection. The algorithm evaluated and labeled images from the color fundus as normal or having DR, identifying particular events of patient comparison. The work in Dutta et al. (2018) suggested an automated information model that classifies DR’s primary antecedents. This model was equipped with three forms of neural network backpropagation, and Deep Convolutional Neural Network (CNN).

Any retinopathy diagnostics model must measure the weights that give the patient's eye intensity level. The primary difficulty of this analysis is the exact judgment of thresholds for each functional level. Weighted Fuzzy C-means algorithm was used to define target level thresholds. The model should help determine the right degree of seriousness in diabetic retinopathy photos. Orujov et al. (2020) adopted a fuzzy-based edge detection method to segment blood vessels in retinal images, which can be adopted for retinopathy detection. Karthiyayini & Shenbagavadivu (2020) adopted association rule mining for disease diagnostics from retinal images.

The Deep Learning algorithms are effective in evaluating the properties of blood pressure, fluid loss, exudates, hemorrhages, and micro-aneurysms as well as other structural features of biomedical images (Khan et al., 2020; Sahlol et al., 2020; Sahlol et al., 2020). Feature fusion is an important technique employed for pattern recognition in images. Feature fusion can be employed to fuse both manual (handcrafted) features and features extracted from inner layers of deep neural networks (Woźniak & Połap, 2018; Afza et al., 2021; Nisa et al., 2020). The advantages of feature fusion allow to achieve more robust and accurate performance.

This paper aims at the detection of DR determined from retinal fundus images. The contribution of this paper is as follows. At first, the ophthalmoscopic characteristics are derived from the photographs of the retina, as well as extracting local binary sequence, gray-level co-occurrence vector, run length, and adding certain morphological operations.

The novelty of our proposed method is the use of the SMO classification algorithm, which has not been done before for DR recognition.

The remainder of this paper is structured as follows. The “Materials and methods” section described the datasets used and the methodology applied. The “Results” section evaluates the results of the proposed methods. The “Conclusion” section summarizes the paper.

## Materials and Methods

This section explicitly details the processing of the diabetic retinopathy through the fusion of features from gray level co-occurrence matrix (GLCM) and gray level run length matrix (GLRLM) and Continuous Ridgelet Transform (CRT). Specific problems regarding feature extraction approaches are examined, and the proposed scheme is refined.

### Materials

The data image collection used for this analysis consists of photographs previously identified as normal (without DR) images and abnormal (DR) images with different stages like mild, moderate, and severe. Two openly accessible datasets such as DIARETDB1 (DIARETDB1, 2007) and KAGGLE (Kaggle Dataset, 2019) are collected. The DIARETDB1 contains 89 fundus color images (size of 1500 × 1152). According to the experts who partake in the assessment, 84 images are assigned as DR, and the remaining 5 images are normal. The fundus images were taken using the identical optical fundus camera with different exposure settings for a similar 50-degree field of view. This collection of data is referred to as "fundus images calibration stage 1."

The other retina data is extracted from Kaggle, which are retina scan images at APTOS 2019 Blindness Detection dataset. These images have a size of 224 × 224 pixels so that they can be conveniently used with several pre-trained neural network models. This dataset contains 5 categories of colored fundus images: No DR, Mild, Moderate, Severe, and PDR.

### Methods

**Figure 3:**
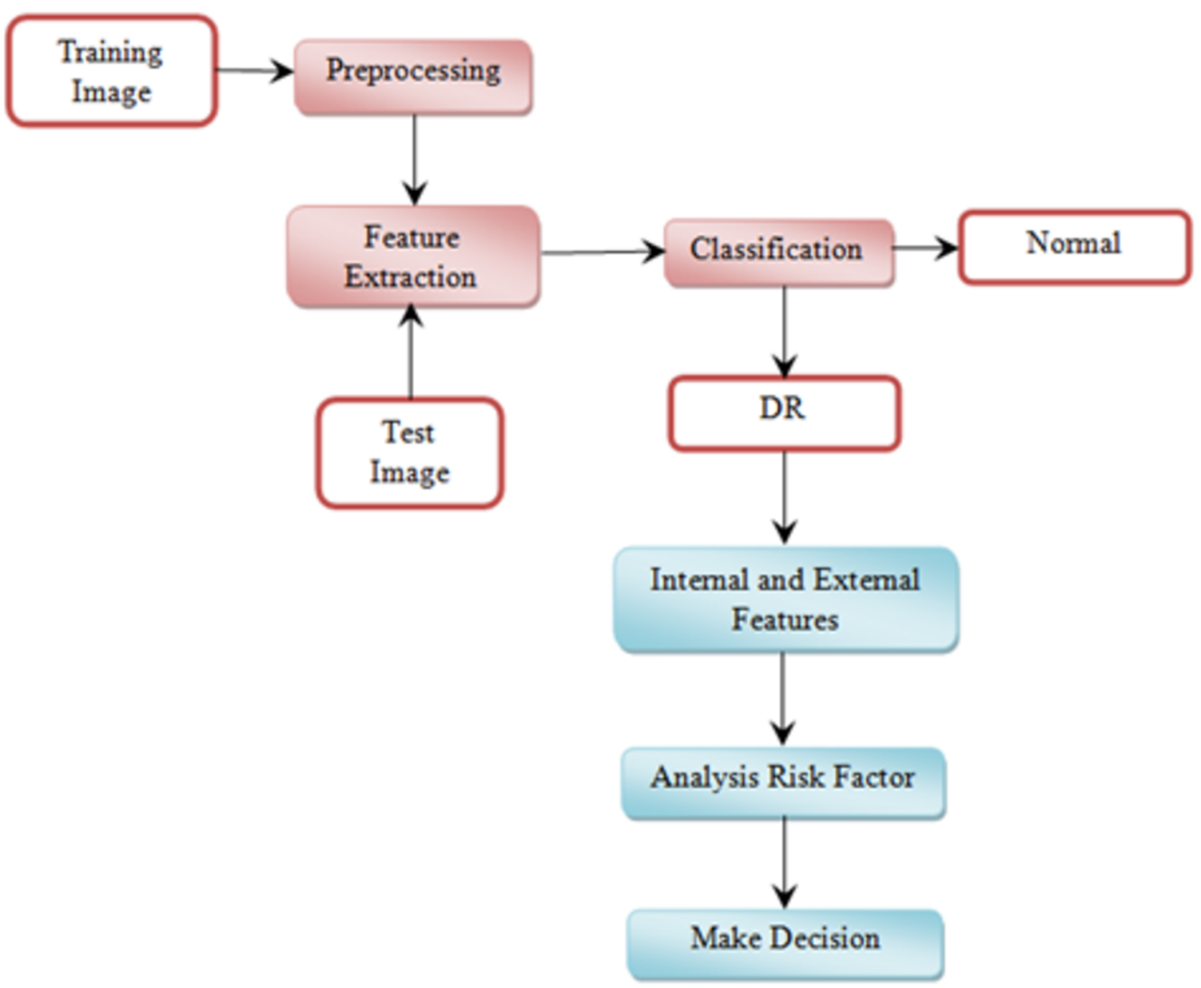
Overview of the proposed methodology workflow.

This section explains the overall general workflow (shown in Fig. 3), and also explains the features used for detecting DR. Premature clinical symptoms of diabetic retinopathy consist of microaneurysms, hemorrhages of dots, spots of cotton wool, blots, and intraretinal microvascular anomalies (IRMAs). Table 1 shows the clinical features of DR.

**Table 1:** Ophthalmoscopic features for retina disease symptoms.

Retina disease symptoms	Ophthalmoscopic features
No retinopathy Mild Moderate Severe Proliferative diabetic retinopathy	- Microaneurysms Retinal hemorrhage, Hard exudates Hemorrhage, venous beading, intraretinal microvascular anomalies Neovascularization, vitreous hemorrhage

Microaneurysms and dot hemorrhages occur on the fundus as small lesions that characterize the ballooning of capillaries in which the vessel wall is weakened by the lack of pericyte protection and/or glial attachment. Hemorrhages and fluid release from microaneurysms contribute to intermittent edema which may leave heavy deposits of lipoproteins (“exudates”) in the retinal neuropile before reabsorbed (Lechner, O’Leary & Stitt, 2017).

### Grey level co-occurrence matrix (GLCM)

The GLCM defines the texture relationship between pixels by executing an action in the images based on the second-order statistics. For this operation, normally two pixels are used. The GLCM calculates the frequency defined by the variations of these pixel intensity values, which reflects the pixel-pair occurrence creation (Sastry et al., 2012). The GLCM features are described as a matrix having the same number of rows and columns as the grey features in the image. Based on their location, all pixel pairs can differ. Such matrix components include the mathematical probability values of second-order, based on the rows and columns gray color. The transient matrix is very big if the intensity values are large (Mohanaiah, Sathyanarayana & GuruKumar, 2013). The GLCM size depends on the values of gray level retained by an image.

Assign Img be an image with N gray levels, an N-by-N dimensional matrix would be the GLCM for the image. At position (i,j), this GLCM tracks the number of times two levels of intensity i and j co-occur at orientation θ in the image Img at distance d. The GLCM of an image Img with rows, columns and offset (x,y), can be characterized as

(1) $$GLCM_{x,y\left(i,j\right)}=\sum_{a=1}^{row}\sum_{b=1}^{col}\begin{array}{c} 1,\;if\;Img\left(a,b\right)=iand\;Img\left(a+x,b+y\right)=j\\ 0,\;otherwise \end{array},$$

GLCM is expected to keep the probability of co-occurrence of any two intensities, rather than the count. And the GLCM values are translated to show probabilities. To that effect, to determine estimates, the number of times a given mixture of intensities occurs is determined by the overall number of potential results. A GLCM is converted into approximate probabilities as follows:

(2) $$P\left(i,j\right)=\frac{coInten_{i,j}}{\sum_{i=1}^{gr}\sum_{j=1}^{gr}coInten_{i,j}}$$

here i and j is the row and column, $coInten_{i,j}$ represents the count of co-occurrences of intensity values i and j, and gr is the total number of intensity values (Do-Hong, Le-Tien & Bui-Thu, 2010).

The GLCM features used in this work are: autocorrelation, correlation, cluster shade, cluster prominence, contrast, difference entropy, dissimilarity, difference variance, energy, entropy, homogeneity, information measure of correlation, maximum probability, inverse difference, sum of average, sum of entropy, sum of squares variance, and sum of variance.

### Gray level run length matrix (GLRLM)

GLRLM is a model representing texture that finds out the spatial plane characteristics of each pixel using high-order statistics. In GLRLM, statistics involved are the number of gray level value pairs and their length of runs in a region of interest (ROI). A gray level run is a group of pixels with the same value of the gray level, spread in the ROI in consecutive and collinear directions. The number of pixels is the length of gray level run in that particular set. Therefore, such a set is defined by a gray level value and the length of a gray level running mutually. GLRLM is a type of two-dimensional histogram in the structure of a matrix that records all the different combinations of gray level values and gray level.

The gray level values and runs are conventionally indicated as row and column keys, respectively, of the matrix, thus the (i,j)-th matrix value determines the count of combinations whose gray level value is i and whose run length is j. Four major directions are typically known, i.e., horizontal (0°), vertical (90°), diagonal (135°), and anti-diagonal (45°).

Suppose $P_{ij}$ is the (i,j)-th GLRLM point. Additionally, $N_{r}$ is used to indicate the set of dissimilar run lengths that currently exist in the ROI, and $N_{g}$ is used to indicate the set of different gray shades. Then at last N be the cumulative number of pixels in the ROI.

(3) $$N=\sum_{i\in N_{g}}\sum_{j\in N_{r}}jP_{i,j}$$

Table 2 shows the formulas for the GLRLM features, where:

(4) $$N_{ve}=\sum_{i\in N_{g}}\sum_{j\in N_{r}}P_{ij}$$

**Table 2:** Summary of gray-level run length matrix (GLRLM) features.

GLRLM features	Formula
Short Run Emphasis (SRE)	$\sum_{i\in N_{g}}\sum_{j\in N_{r}}\frac{P_{ij}}{j^{2}}/N_{ve}$
Long Run Emphasis (LRE)	$\sum_{i\in N_{g}}\sum_{j\in N_{r}}j^{2}P_{ij}/N_{ve}$
Gray Level Non-uniformity (GLN)	$\sum_{i\in N_{g}}{\left(\sum_{j\in N_{r}}P_{ij}\right)}^{2}/N_{ve}$
Run Length Non-uniformity (RLN)	$\sum_{j\in N_{r}}{\left(\sum_{i\in N_{g}}P_{ij}\right)}^{2}/N_{ve}$
Run Percentage (RP)	$\sum_{i\in N_{g}}\sum_{j\in N_{r}}P_{ij}/N$
Low Gray Level Run Emphasis (LGRE)	$\sum_{i\in N_{g}}\sum_{j\in N_{r}}\frac{P_{ij}}{i^{2}}/N_{ve}$
High Gray Level Run Emphasis (HGRE)	$\sum_{i\in N_{g}}\sum_{j\in N_{r}}i^{2}P_{ij}/N_{ve}$
Short Run Low Gray Level Emphasis (SRLGE)	$\sum_{i\in N_{g}}\sum_{j\in N_{r}}\frac{P_{ij}}{i^{2}j^{2}}/N_{ve}$
Short Run High Gray Level Emphasis (SRHGE)	$\sum_{i\in N_{g}}\sum_{j\in N_{r}}\frac{i^{2}P_{ij}}{j^{2}}/N_{ve}$
Long Run Low Gray Level Emphasis (LRLGE)	$\sum_{i\in N_{g}}\sum_{j\in N_{r}}\frac{j^{2}P_{ij}}{i^{2}}/N_{ve}$
Short Run High Gray Level Emphasis (LRHGE)	$\sum_{i\in N_{g}}\sum_{j\in N_{r}}i^{2}j^{2}P_{ij}/N_{ve}$

### Continuous ridgelet transform (CRT)

The idea of the latter is to display linear features image to point using Radon transform and the subsequent use of wavelet transformations. The result of this operation is an effective representation of two-dimensional functions with piecewise smooth areas separated by linear plots. The main difference between ridge functions and wavelet functions are that ridgelets are two-dimensional inseparable functions and determine not only the parameters of scale and shift but also their orientation in space (Candès, 1999). The CRT of function f(x) is defined as

(5) $$R(a,b,\theta )=\int \psi_{a,b,\theta }(x)f(x)dx$$

where $\psi_{a,b,\theta }$ are the ridgelets defined by

(6) $$\psi_{a,b,\theta }(x)=a^{-1/2}\psi ((x_{1}\cos\theta +x_{2}\sin\theta -b)/a)$$

here, ψ(⋅) is the smoothly decaying function.

In images, CRT can be calculated via Radon transform (RT). RT of a two-dimensional object f is the set of line integrals indexed by (θ,t)∈[0,2π) given by

(7) $$R_{f}(\theta ,t)=\int f(x_{1},x_{2})\delta (x_{1}\cos\theta +x_{2}\sin\theta -t)dx_{1}dx_{2}$$

where δ is the Dirac distribution. Then CRT applies a 1-D wavelet transform to the projections of the RT as follows:

(8) $$R(a,b,\theta )=\int R_{f}(\theta ,t)a^{-1/2}\psi (t-b/a)dt$$

**Figure 4:**
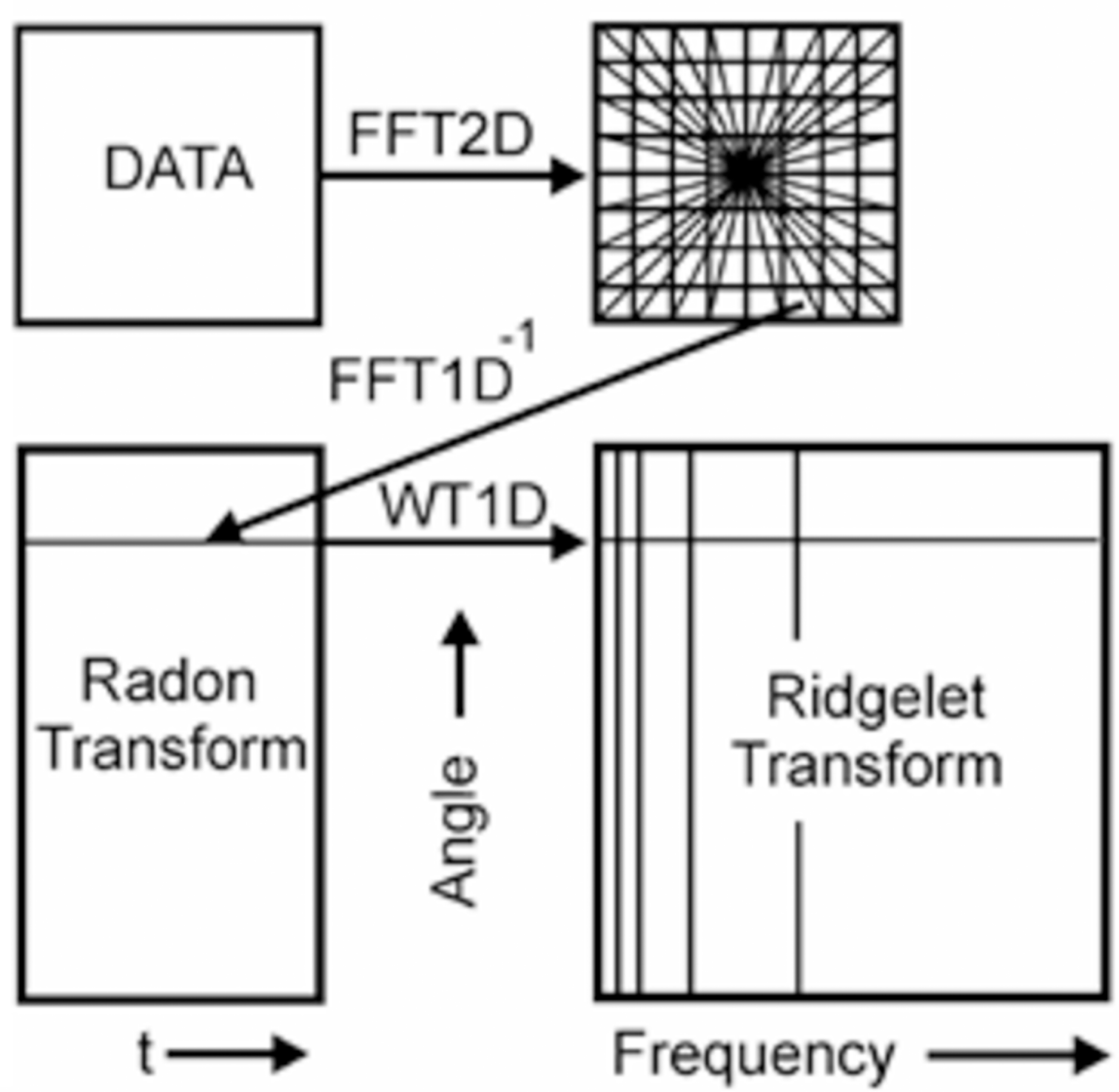
A schematic representation of Continuous Ridgelet transform.

The Ridglet transform scheme is shown in Fig. 4. The main stages of its implementation are as follows:

1. Calculation of direct two-dimensional transformation Fourier (FFT2D).
2. The application of forward Fourier transform from the rectangular grid of coordinates to the polar grid using the interpolation operation coefficients of the Fourier transform.
3. The use of the inverse one-dimensional transform Fourier (IFFT1D) to each line of the obtained polar Noah grid. The result of this operation is the Radon transform coefficients.
4. Application to the plane of the Radon transform of the one-dimensional wavelet transform (WT1D) along with a variable that determines the angle of the line produces the ridgelet coefficients.

### Diabetic retinopathy detection

**Figure 5:**
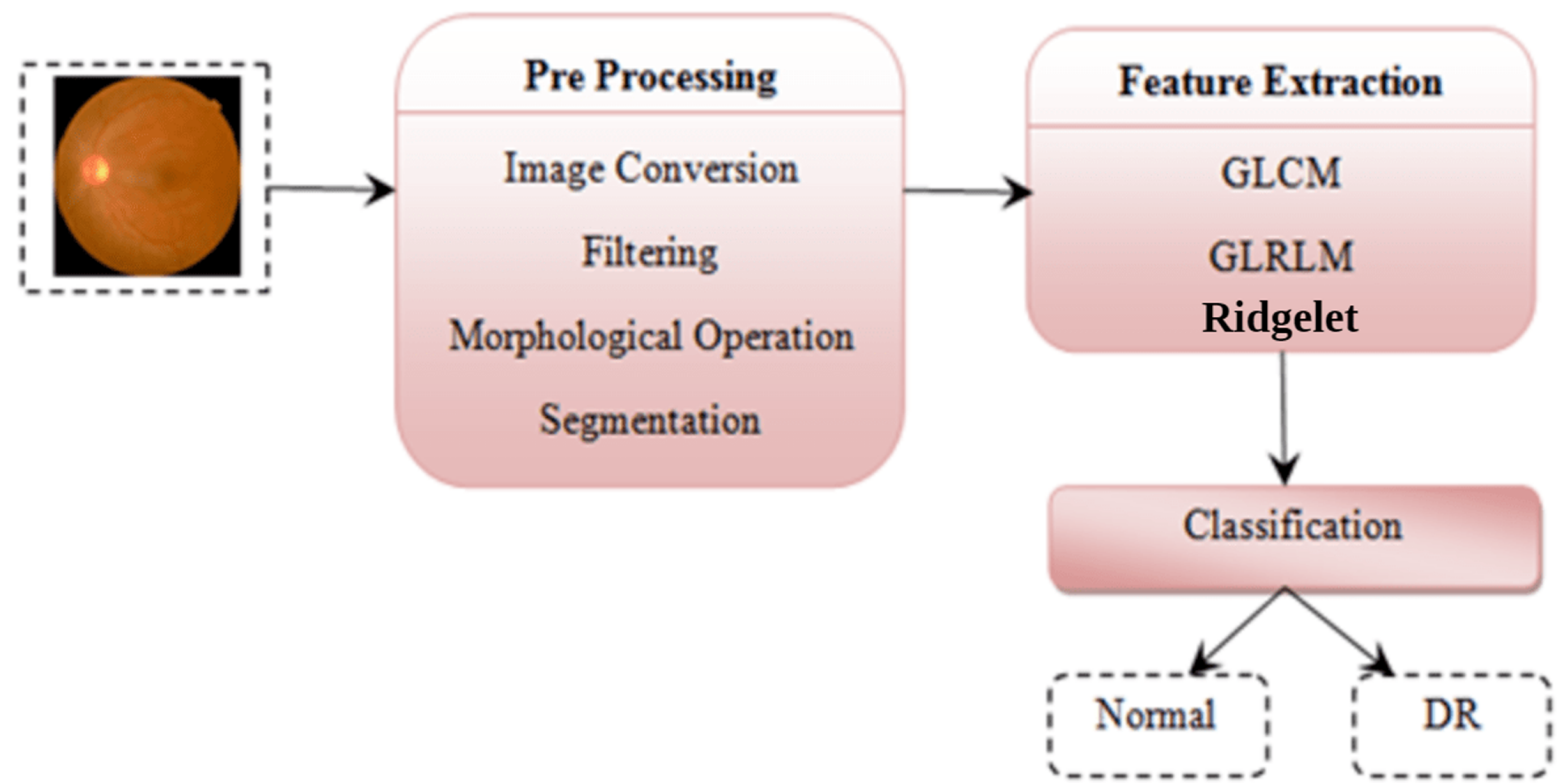
Diabetic retinopathy detection architecture. Image credit: DIARETDB1. © Tomi Kauppi, Valentina Kalesnykiene, Joni-Kristian Kamarainen, Lasse Lensu, Iiris Sorri, Asta Raninen, Raija Voutilainen, Juhani Pietilä, Heikki Kälviäinen, and Hannu Uusitalo.

This section explains the proposed methodology for diabetic retinopathy detection. Figure 5 shows the proposed diabetic retinopathy detection.

**Figure 6:**
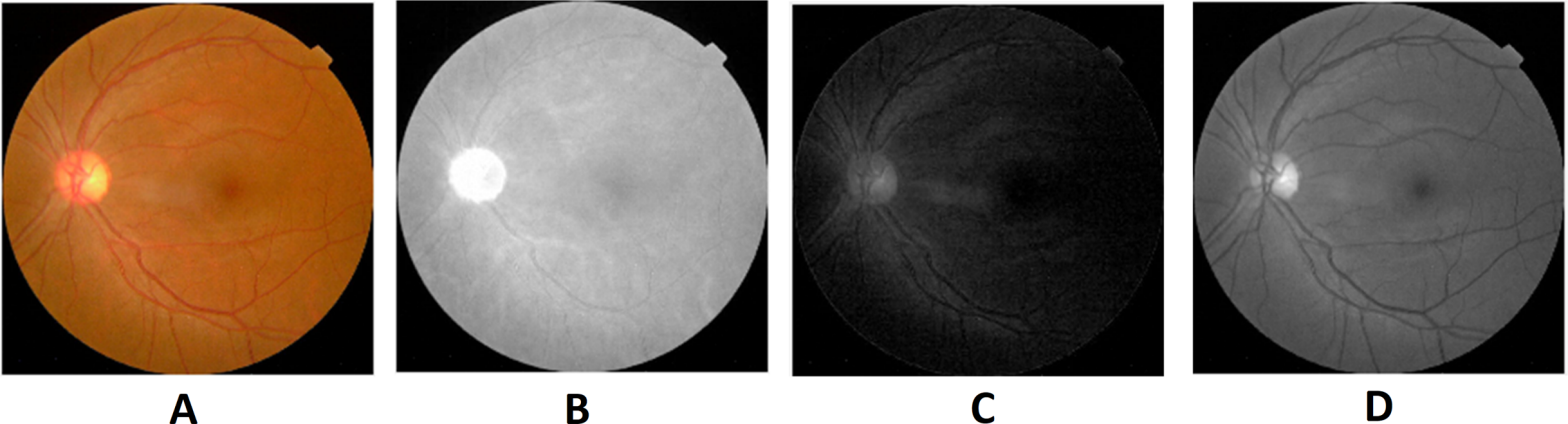
Red, green and blue (RGB) color channels of a fundus image: (A) input image, (B) red channel, (C) green channel, (D) blue channel. Image credit: DIARETDB1. © Tomi Kauppi, Valentina Kalesnykiene, Joni-Kristian Kamarainen, Lasse Lensu, Iiris Sorri, Asta Raninen, Raija Voutilainen, Juhani Pietilä, Heikki Kälviäinen, and Hannu Uusitalo.

Preprocessing is the initial step of the image processing techniques. It is used to enhance the image quality which gives clear visualization. The preprocessing consists of image conversion, filtering, morphological operation, and segmentation. The retina images provided by ophthalmologists in the public repositories are shown in color format. The retina image is divided into the following channels in the image conversion step: Red, Green, and Blue (Fig. 6). The green channel image is taken for the next process due to the high contrast.

Image filtering is used to denoising the image. It is the method of eliminating noise from the retina images. Laplacian filter is used for image denoising. The Laplacian of an image shows regions with an accelerated change in intensity and is an example of a second-order or second type of enhancement derivatives. The discovery of the fine details of an image is especially good. A Laplacian operator can improve any function that has a sharp discontinuity. The Laplacian L(x,y) of an image with pixel intensity I(x,y) is defined as follows:

(9) $$L\left(x,y\right)=\frac{\partial^{2}I}{\partial x^{2}}+\frac{\partial^{2}I}{\partial y^{2}}$$

**Figure 7:**
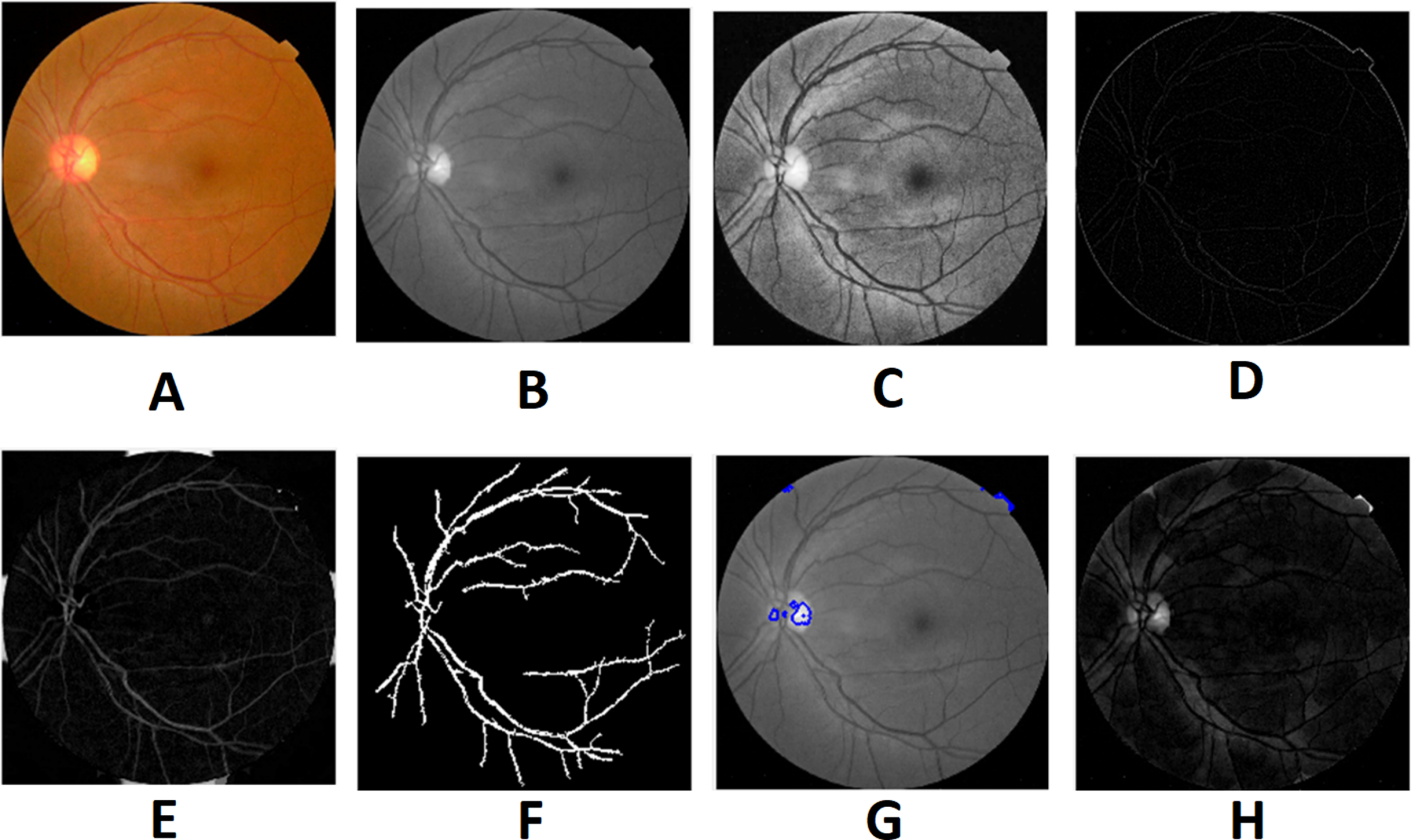
Preprocessed result of normal retina image: (A) input image, (B) green channel, (C) histogram enhanced, (D) filtered image, (E) after bottom hat transform, (F) after top hat transform, (G) blood vessels segmented, (H) contours enhanced. Image credit: DIARETDB1. © Tomi Kauppi, Valentina Kalesnykiene, Joni-Kristian Kamarainen, Lasse Lensu, Iiris Sorri, Asta Raninen, Raija Voutilainen, Juhani Pietilä, Heikki Kälviäinen, and Hannu Uusitalo.

**Figure 8:**
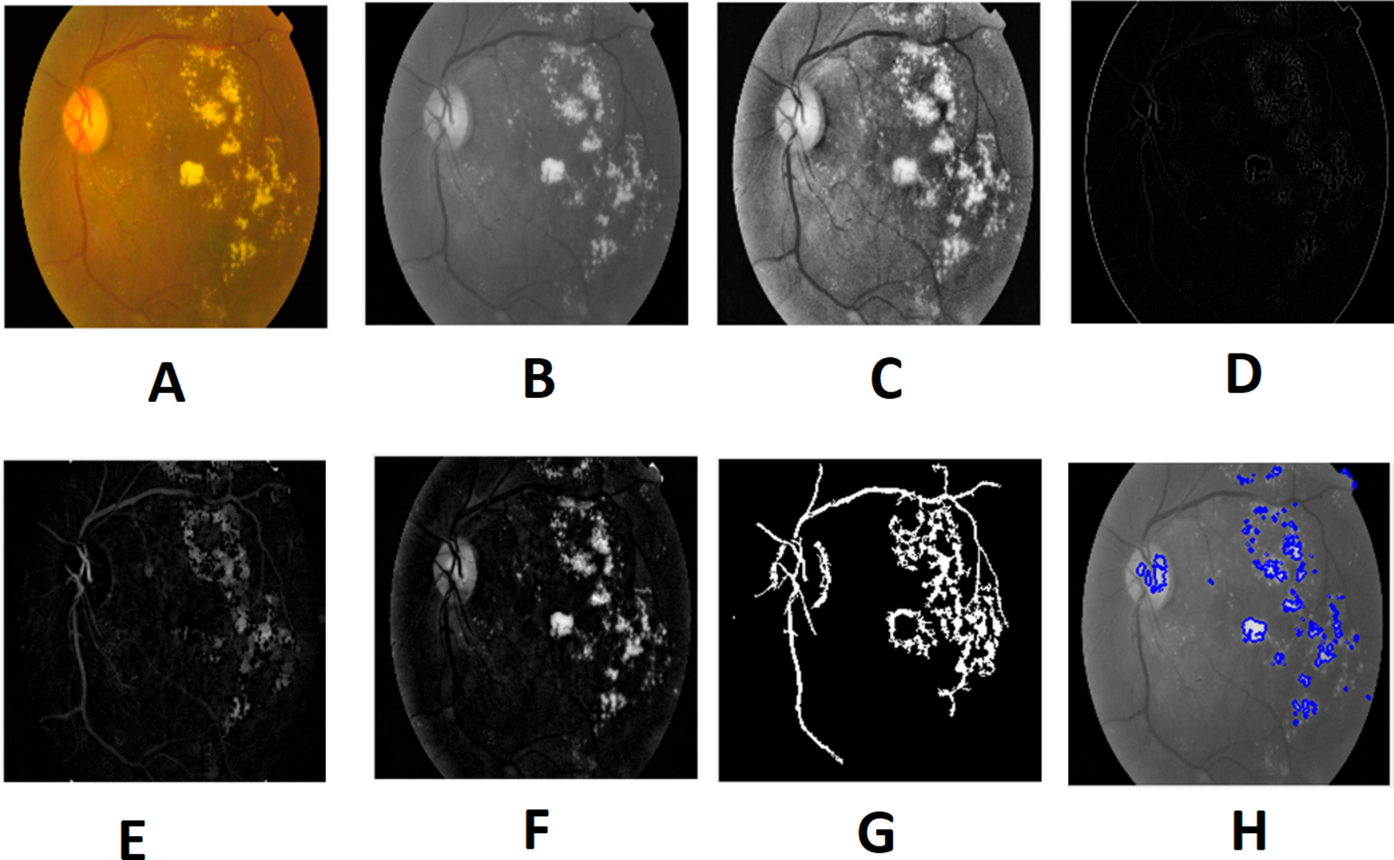
Preprocessed result of diabetic retinopathy image: (A) input image, (B) green channel, (C) histogram enhanced, (D) filtered image, (E) after bottom hat transform, (F) after top hat transform, (G) blood vessels segmented, (H) contours enhanced. Image credit: DIARETDB1. © Tomi Kauppi, Valentina Kalesnykiene, Joni-Kristian Kamarainen, Lasse Lensu, Iiris Sorri, Asta Raninen, Raija Voutilainen, Juhani Pietilä, Heikki Kälviäinen, and Hannu Uusitalo.

Morphological processing of images is a range of non-linear operations associated with the structure or morphology of features in an image. This technique investigates an image called a structuring feature with a specific outline or blueprint. The structuring factor is located in the picture at all possible positions and is contrasted with the respective pixel neighborhood (Indumathi & Sathananthavathi, 2019). A morphological closure procedure is performed to clear the main blood vessels. Next, we perform binarization and noise reduction by setting a threshold to remove the isolated pixels. The morphological top and bottom hat transform are applied for basic segmentation. The top-hat transform can be used to improve contrast with non-uniform illumination in a grayscale setting. The transform will distinguish tiny light objects in an image, too. The transformation of the bottom hat can be used to locate size pits in a grayscale image. Binarization is used to identify blood vessels and regions of the candidate (1), and other background regions (0). By eliminating the isolated pixels with a neighborhood associated value below 25, the noise components that appear identical to MAs can be effectively eliminated from the binarized image. Figures 7 and 8 show the preprocessing result of normal and DR images.

### Feature extraction

Texture in feature extraction is the key characteristic of an image. Numerous methods for texture analysis are introduced in various fields of study. We use a fusion of textural GLCM and GLRLM features and Ridgelet Transform features.

### Image classification

This is the last stage of the recognition process in diabetic retinopathy disease. After extraction of features, the retina fundus image is classified as normal, or DR. SMO (Sequential Minimal Optimization) is a straightforward algorithm that uses only two Lagrange multipliers at each iteration to move the chunking process to the nearest possible expression. It determines the optimal value for these multipliers and updates the SVM until it fixes the whole QP problem. The benefit of SMO is that the optimization sub-problem can be solved analytically with two Lagrange multipliers.

Detection of the diagnostic induced disease has its limits. When a device is prepared for a classification task, the issues are different. It would be able to work automatically by providing the system with proper classification instructions, which will have better classification performance. This study uses the SMO algorithm for classifying the DR.

## Experimental Results

### Performance measures

The performance analysis of the proposed system is explained in this section. The DR detection is implemented using MATLAB 2019b (MathWorks Inc., MA, USA). This work is evaluated based on Sensitivity, Specificity, Accuracy and F-score computed as follows:

$$Sensitivity\;(SE)=\frac{TP}{TP+FN}*100\%$$

(10) $$Specificity\;(SP)=\frac{TN}{TN+FP}*100\%$$

$$Accuracy(ACC)=\frac{TP+TN}{N}*100\%$$

$$F-score(F)=\frac{2TP}{2TP+FP+FN}$$

Here TP is a count of true positive class (normal retina), TN is the count of true negative class (DR). FP is the count of false-positive (normal retina predicted as DR). FN is the count of false-negative class (DR is predicted as the normal retina).

## Results and Comparison

**Figure 9:**
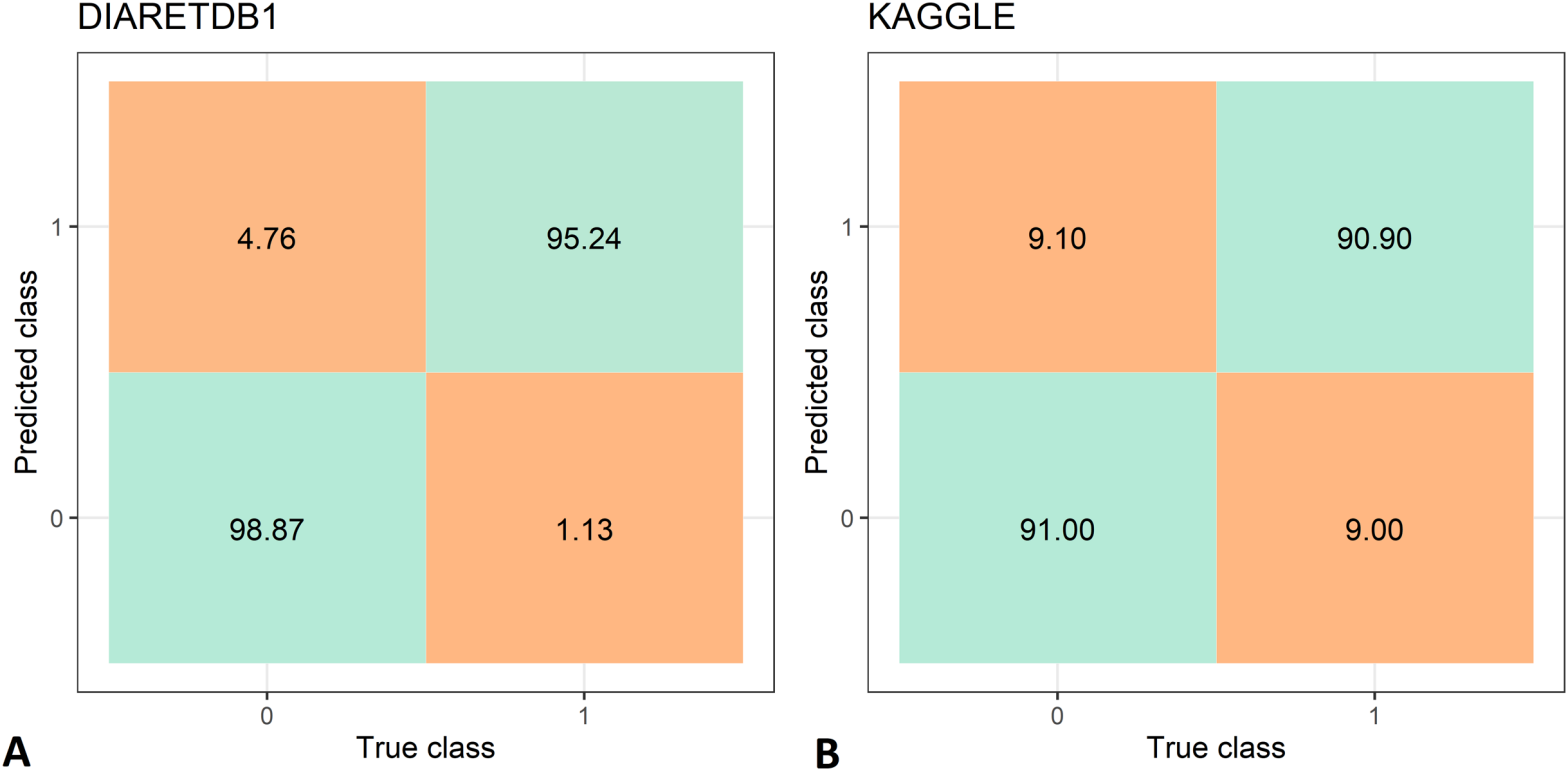
Confusion matrices for diabetic retinopathy recognition: (A) DIARETDB1 dataset, (B) KAGGLE datasets.

The feature extraction time of the two data set is shown in Tab. 3, while the DR recognition results are shown in Tab. 4. The feature extraction time from the DIARETDB1 and KAGGLE databased is about 2–2.5 min., which make the proposed method usable for real-time clinical applications. The proposed method has achieved an accuracy of 97.05%, sensitivity of 98.87%, and specificity 95.24% on the DIARETDB1 dataset. On the KAGGLE dataset, the proposed method achieved an accuracy of 91.0%, sensitivity of 90.9%, and specificity of 91.0%. The results for both datasets are summarized as classification confusion matrices in Fig. 9.

**Table 3:** Feature extraction time for DIARETDB1 and KAGGLE datasets.

Dataset	Feature extraction time (s)
DIARETDB1	131.56
KAGGLE	159.19

**Table 4:** Performance evaluation metrics for DIARETDB1 and KAGGLE datasets.

Dataset	SE (%)	SP (%)	ACC (%)	F-score
DIARETDB1	98.87	95.24	97.05	0.969
KAGGLE	90.9	91.0	91.0	0.909

We compare our results with the results of other authors, which used a wide variety of techniques from handicraft feature extraction and heuristic optimization methods to deep learning networks, achieved on the same datasets. The comparison is presented in Tabs. 5 and 6. Other authors have employed a wide variety of machine learning, deep learning and heuristic optimization techniques.

**Table 5:** Comparison of performance evaluation results for DIARETDB1 dataset.

Reference	Method	SE (%)	SP (%)	ACC (%)
(Das, Dandapat & Bora, 2019)	Contrast sensitivity index (CSI), Shannon entropy, multi-resolution (MR) inter-band eigen features and intra-band energy	–	–	85.22
(Long et al., 2019)	Fuzzy C-means clustering (FCM) & support vector machine (SVM)	97.5	97.8	97.7
(Mateen et al., 2020)	Feature fusion from Inception-v3, ResNet-50, and VGGNet-19 models	–	–	98.91
(Pruthi, Khanna & Arora, 2020)	Glowworm Swarm optimization	–	–	96.56
(Sharif et al., 2020)	Histogram orientation gradient (HOG) and local binary pattern (LBP) feature fusion & decision tree (DT)	98.1	91.8	96.6
(Zago et al., 2020)	Custom convolutional neural network (CNN)	90	87	–
(Chetoui & Akhloufi, 2020)	Extended Inception-Resnet-v2 network fine-tuned by cosine annealing strategy	98.8	90.1	97.1
(Alaguselvi & Murugan, 2020)	Morphological operation, matched filter, principal component analysis (PCA), edge detection by ISODATA, and convex hull transform	99.03	98.37	98.68
**Proposed**	**A fusion of texture and ridgelet features & SMO**	**98.87**	**95.24**	**97.05**

**Table 6:** Comparison of performance evaluation results for KAGGLE dataset.

Reference	Method	SE (%)	SP (%)	ACC (%)
(Bodapati et al., 2020)	ConvNet features & deep neural network (DNN)	–	–	80.96
(Li et al., 2019)	DCNN features + SVM	–	–	86.1
(Mateen et al., 2019)	VGG-19 features, singular value decomposition (SVD)	–	–	98.34
(Nazir et al., 2019)	Tetragonal local octal patterns & extreme learning machine (ELM)	–	–	99.6
(Qummar et al., 2019)	Ensemble of Resnet50, Inceptionv3, Xception, Dense121, Dense169 models	–	95	80.8
(Sudha & Ganeshbabu, 2021)	VGG-19 model, structure tensor and active contour approximation	98.83	96.76	98.28
(Vaishnavi, Ravi & Anbarasi, 2020)	Contrast-limited adaptive histogram equalization (CLAHE) model and AlexNet architecture with SoftMax layer	92.00	97.86	95.86
(Math & Fatima, 2020)	Custom CNN model with fine-tuning	96.37	96.37	–
**Proposed**	**A fusion of texture and ridgelet features & SMO**	90.9	91.0	91.0

On the DIARETDB1 dataset, (Das, Dandapat & Bora, 2019) used contrast sensitivity index (CSI), Shannon entropy, multi-resolution (MR) inter-band eigen features and intra-band energy. (Long et al., 2019) used Fuzzy C-means clustering (FCM) and SVM classifier. (Mateen et al., 2020) employed feature fusion from Inception-v3, ResNet-50, and VGGNet-19 deep convolutional models. (Pruthi, Khanna & Arora, 2020) used a nature-inspired Glowworm Swarm Optimization algorithm. (Sharif et al., 2020) adopted Histogram orientation gradient (HOG) and local binary pattern (LBP) feature fusion combined with Decision Tree (DT) classifier. (Zago et al., 2020) used a custom fully patch-based CNN. Chetoui and (Chetoui & Akhloufi, 2020) used an extended Inception-Resnet-v2 network fine-tuned by cosine annealing strategy. Alaguselvi and (Alaguselvi & Murugan, 2020) used morphological operation, matched filter, Principal Component Analysis (PCA), edge finding by ISODATA, and convex hull transform. On the KAGGLE dataset, (Bodapati et al., 2020) used ConvNet features and Deep Neural Network (DNN) for classification. (Li et al., 2019) employed DCNN as feature extractors and SVM for classification. (Mateen et al., 2019) used pretrained CNN model (Inception-v3, Residual Network-50, and Visual Geometry Group Network-19) feature fusion followed by the softmax classifier. (Nazir et al., 2019) used tetragonal local octal patterns and Extreme Learning Machine (ELM). (Qummar et al., 2019) used an ensemble of Resnet50, Inceptionv3, Xception, Dense121, and Dense169 deep network models. (Sudha & Ganeshbabu, 2021) adopted the VGG-19 model combined with structure tensor for enhancing local patterns of edge elements and active contours approximation for lesion segmentation. (Vaishnavi, Ravi & Anbarasi, 2020) used Contrast-limited adaptive histogram equalization (CLAHE) model and AlexNet network architecture with SoftMax layer for classification. (Math & Fatima, 2020) used a custom CNN architecture with fine-tuning.

Our results demonstrate the competitiveness of our method with the state-of-the-art. On the DIARETDB1 dataset, our method achieved very good sensitivity, while considering the accuracy, only the methods of (Long et al., 2019), and (Alaguselvi & Murugan, 2020) have achieved marginally higher accuracy. However, for disease diagnostics, sensitivity is more important than accuracy (Loong, 2003). For the KAGGLE dataset, our method has performed slightly worse, but still achieved an accuracy over 90%, which is in line with other state-of-the-art DR recognition methods.

## Discussion

The proposed method for the detection of diabetic retinopathy using the Ridgelet Transform and the Sequential Minimal Optimization (SMO) presents an alternative to recent works based on convolutional networks and deep learning. The achieved results are competitive with the state-of-the-art results while the common pitfalls of deep learning methods such as the need for very large datasets for training deep network models as well as the underfitting and overfitting problems are avoided. Moreover, the results provided by artificial intelligence methods are not explainable. As a result, any black box diagnostics systems are not accepted by a professional ophthalmologist in the real world, regardless of their fine results.

The method presented in this article adopts a traditional approach. However, our approach is different from other works based on feature creation and classification. The proposed method also has some limitations as using all textural and Ridgelet features may include irrelevant features for the task of DR recognition, which can incur larger computation time, and sometimes even reduce the recognition accuracy. These limitations could be overcome by further fine-tuning the parameters of SMO technique.

## Conclusion

The integration of the extracted features using texture analysis methods (GLCM and GLRLM) and Ridgelet Transform features suggests an automated approach for classifying Diabetic Retinopathy (DR). The extracted features using the suggested approach are used for the process of classification using the SMO classifier to identify DR. The results show that the proposed method is competitive with other state-of-the-art methods on the DIARETDB1 and KAGGLE datasets (we achieved 98.87% sensitivity, 95.24% specificity, 97.05% accuracy on DIARETDB1 dataset, and 90.9% sensitivity, 91.0% specificity, 91.0% accuracy on KAGGLE dataset). The obtained results show that image processing techniques combined with optimization methods can still be competitive to convolutional network and deep learning based approaches.

## Supplemental Information

10.7717/peerj-cs.456/supp-1 Supplemental Information 1 Code describing the implementation of the proposed method. Code of implemented method (in MATLAB).

10.7717/peerj-cs.456/supp-2 Supplemental Information 2 Features extracted from DIARETDB1 dataset.

10.7717/peerj-cs.456/supp-3 Supplemental Information 3 Features extracted from KAGGLE dataset.

## Additional Information and Declarations

### Data Availability

The following information was supplied regarding data availability: The DIARETDB1 dataset is available at the Lappeenranta University of Technology: http://www2.it.lut.fi/project/imageret/diaretdb1/ The Diabetic Retinopathy Detection dataset is available at Kaggle: https://www.kaggle.com/c/diabetic-retinopathy-detection Data and code are available in the Supplemental Files.
